# Readmission prediction via deep contextual embedding of clinical concepts

**DOI:** 10.1371/journal.pone.0195024

**Published:** 2018-04-09

**Authors:** Cao Xiao, Tengfei Ma, Adji B. Dieng, David M. Blei, Fei Wang

**Affiliations:** 1 AI for Healthcare, IBM Research, Cambridge, MA, United States of America; 2 IBM T.J. Watson Research Center, Yorktown Heights, NY, United States of America; 3 Department of Computer Science, Columbia University, New York, NY, United States of America; 4 Weill Cornell Medical School, Cornell University, New York, NY, United States of America; National University of Defense Technology, CHINA

## Abstract

**Objective:**

Hospital readmission costs a lot of money every year. Many hospital readmissions are avoidable, and excessive hospital readmissions could also be harmful to the patients. Accurate prediction of hospital readmission can effectively help reduce the readmission risk. However, the complex relationship between readmission and potential risk factors makes readmission prediction a difficult task. The main goal of this paper is to explore deep learning models to distill such complex relationships and make accurate predictions.

**Materials and methods:**

We propose CONTENT, a deep model that predicts hospital readmissions via learning interpretable patient representations by capturing both local and global contexts from patient Electronic Health Records (EHR) through a hybrid Topic Recurrent Neural Network (TopicRNN) model. The experiment was conducted using the EHR of a real world Congestive Heart Failure (CHF) cohort of 5,393 patients.

**Results:**

The proposed model outperforms state-of-the-art methods in readmission prediction (e.g. 0.6103 ± 0.0130 vs. second best 0.5998 ± 0.0124 in terms of ROC-AUC). The derived patient representations were further utilized for patient phenotyping. The learned phenotypes provide more precise understanding of readmission risks.

**Discussion:**

Embedding both local and global context in patient representation not only improves prediction performance, but also brings interpretable insights of understanding readmission risks for heterogeneous chronic clinical conditions.

**Conclusion:**

This is the first of its kind model that integrates the power of both conventional deep neural network and the probabilistic generative models for highly interpretable deep patient representation learning. Experimental results and case studies demonstrate the improved performance and interpretability of the model.

## Introduction

A hospital readmission is defined as the admission to a hospital within a short amount of time after discharge, where 30-day is typically considered a clinically meaningful time window [1]. Excessive hospital readmissions disrupt the normality of patients’ lives and have negative impacts on the healthcare systems [2]. For example, in the US, it has been reported by the Medicare Payment Advisory Committee that 17.6% of hospital-admitted patients were readmitted within 30 days of discharge, which accounted for $17:9 billion Medicare spending per year, while 76% of them are potentially avoidable [1]. To curb hospital readmission rates, the Patient Protection and Affordable Care Act was set up to penalize hospitals with excessive readmission at a minimum of 3% of their Medicare reimbursement. Despite the efforts, it is estimated that the scrutiny of readmission rates will continue to grow over the next few years.

To prevent excessive readmissions, procedures such as patient follow-ups and educations have been implemented, which could be costly for individual patient. Therefore, targeted follow-ups that focus on patients with high risks of readmissions are preferred. This raises the demand for assessing patient readmission risks and consequently brings the readmission prediction to the forefront of healthcare research. Accurate prediction of hospital readmission is difficult because of its complex entanglements with the patients’ health conditions, especially the chronic ones. In recent years, there have been some research on hospital readmission prediction from patient Electronic Health Records (EHRs) [1–6]. There are many challenges for working with EHR such as its incompleteness, noisiness, heterogeneity, etc. [7], and the existing research typically needs to rely on appropriate feature engineering [4, 8], whose optimality is difficult to justify from both computational and clinical perspectives.

In order to solve the challenges, we seek for deep learning models to perform readmission predictions. Deep learning models are well known for their end-to-end learning capabilities so we do not need to worry about the feature engineering part [9, 10]. Moreover, deep learning models are proved to be very powerful at distilling the complicated relationships hidden in the data and thus demonstrate good prediction performance [10, 11]. In this paper, we develop CONTENT, which is a deep learning model that transforms patients’ complicated event structures in their EHR into deep clinical concept embedding, which can be viewed as a novel form of patient representation encoding the patient clinical conditions from both long and short terms. We draw the analogy between EHR modeling and natural language models [12] to consider the short-term dependencies among medical events in EHRs as local context of a patient journey and long-term effect as global context. Such contexts impact the latent relations between the clinical variables (e.g. diagnoses, procedures, medications, etc.) and the target variable (i.e., readmission). We design a hybrid deep learning model structure that combines topic modelling [13] and Recurrent Neural Network (RNN) [14] to distill the complex knowledge hidden in those contexts and perform accurate readmission prediction.

It is worthwhile to highlight the following aspects of the proposed CONTENT model.

- The proposed model explores both the global and local contexts within the patient journey from his/her EHRs. The global context (the general conditions of the patient, such as those chronic diseases, comorbidities, etc.) is captured by topic models and local context (the short term disease progressions) is captured by RNN. In this way, we can better capture the heterogeneities across different patient individuals and make the model more precise. Empirical results also show the joint modeling could achieve better overall performance evaluated on the readmission prediction tasks.
- Because of the incorporation of the global context, the resultant model is more interpretable comparing to simple RNN models. Our model will produce a context vector for each patient, which characterizes his/her overall condition.

## Background

### Predictive modeling and deep learning

Most of the existing works on predicting 30-day hospital readmissions were developed with administrative claims with certain components from EHR such as vital signs and lab tests [1–3, 5]. Those events are typically aggregated over a certain period of time (a.k.a. observation window) with some simple feature transformation [4, 8], and then fed into a predictor such as logistic regression or random forest for the prediction task [1, 6].

One limitation of those conventional approaches is that they cannot take the time information into account. The temporalities of the events in patient records are crucial because they can potentially suggest the progression pattern of the patient conditions. Recently, researchers have been exploring deep learning models, such as Convolutional Neural Network (CNN) [15, 16] and Recurrent Neural Network (RNN) [17–20] to capture the complex temporal relationships among the medical events. For example, in [15], the authors proposed a multilayered convolutional neural nets (CNNs) to extract complex patient representations that capture convoluted relations among various clinical events. In [16], each patient’s EHR is represented as a temporal matrix with time on one dimension and medical events on the other dimension, and a four-layer CNN model is built for extracting representations. An RNN model was adopted for predicting the onset risk for heart failure patients from their EHRs [17]. A temporal Long Short Term Memory (LSTM, which is a variant of RNN) model is proposed to capture the progression patterns for Parkinson’s disease [18].

These existing works typically construct a unique model for the entire patient cohort. Because of the high heterogeneity of the disease conditions across patient individuals and the complexity of the dependencies of hospital readmission and the medical events within patient EHRs, it would be very difficult to learn a single model that can capture all those complexities with a limited number of patients. The proposed model in this paper assigns a global patient-specific context vector for each patient, and the prediction for the patient is dependent on both the context vector and an RNN. In this way, we can model the patients more precisely.

### RNN and contextual RNN

An RNN is a fully connected neural network with recurrent connections in its hidden layer [10]. They take the input at current time step *t* along with the hidden state at time step t − 1 to compute the current hidden state. Mathematically, an RNN defines the conditional probability of each input *w*_*t*_ given all of the previous inputs *w*_1: *t*−1_ through a hidden state *h*_*t*_ via a softmax function:

$$\begin{array}{c} p\left(w_{t}|w_{1:t-1}\right)≜p\left(w_{t}|h_{t}\right) \end{array}$$

$$\begin{array}{c} h\left(t\right)=f\left(h_{t-1},w_{t-1}\right) \end{array}$$

The function *f*(⋅) can either be a standard RNN cell or a more complex cell such as gated recurrent (GRU) unit [21] or long short-term memory (LSTM) unit [22]. In this paper, we choose the GRU cell for CONTENT model because it can achieve similar effects as LSTM with a much simpler structure. More concretely, GRU can overcome the vanishing gradient problem as well as capture the effect of long-term dependencies with a sophisticated gating mechanism. Given input ***x***_*t*_, the function *GRU*(⋅) updates hidden states as follows.

$$\begin{array}{cc} & z_{t}=\sigma \left(U_{z}x_{t}+W_{z}h_{t-1}\right)\\ & r_{t}=\sigma \left(U_{r}x_{t}+W_{r}h_{t-1}\right)\\ & {\tilde{h}}_{t}=tanh\left(U_{h}x_{t}+r_{t}⊙W_{h}h_{t-1}\right)\\ & h_{t}=z_{t}⊙h_{t-1}+\left(1-z_{t}\right)⊙{\tilde{h}}_{t} \end{array}$$

where *σ*(⋅) denotes the sigmoid function and ⊙ denotes the element wise multiplication; ***x***_*t*_ is the input at time step *t*, ***h***_*t*−1_ is the previous hidden state. ***U***_*z*_ and ***W***_*z*_ are weight matrices for update gate ***z***_*t*_, and ***U***_*r*_ and ***W***_*r*_ are weight matrices for the reset gate *r*_*t*_. We drop the biases here for simplicity of notation. In this formulation, the update gate selects whether the hidden state is updated with a new hidden state ${\tilde{h}}_{t}$. The reset gate ***r***_*t*_ decides whether the previous hidden state ***h***_*t*−1_ is ignored [21].

Although in principle RNN-based models can “remember” arbitrarily long span history if provided enough capacity, in practice such large-scale neural networks can easily encounter difficulties during optimization or overfitting [23, 24]. Thus, several contextual recurrent neural network models were proposed to explicitly model long span context to improve learning [7, 9, 13, 25–27]. In language models, since much of the long span context comes from semantic coherence, and the topic models [13] can be used to capture global semantic coherency. Therefore, the recently proposed TopicRNN model [26] uses topic models in a recognition network to directly capture long-range semantic dependencies (i.e. global context) via latent topics. These latent topics are then used as additional bias to the output layer of an RNN-based model. In this study, CONTENT is an extension of the contextual RNN model, particularly the TopicRNN model, with hierarchical inputs (“hospital visits” and “clinical events”) and sequential binary outputs (indication of readmission) at the “visit” level in EHR data. In addition, the CONTENT does not model stop words.

## Materials and method

### Data description

**Fig 1:**
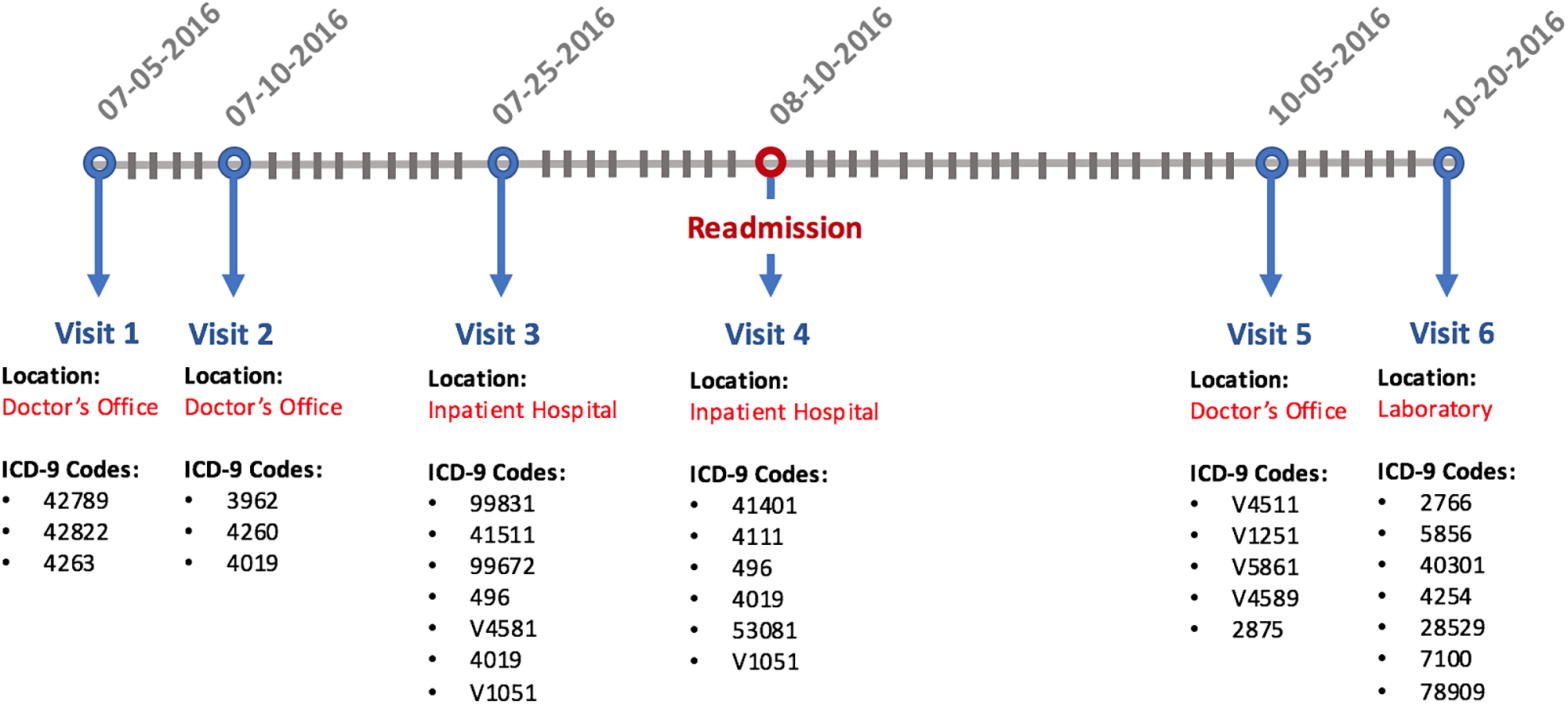
An example segment of EHR records, where visits could occur to different locations. Patients who are re-admitted to “inpatient hospital” within 30 days of their releases from “inpatient hospital” are considered readmissions.

In this work, we conducted the experiment using data from a real world EHR repository of Congestive Heart Failure (CHF) cohort including 5,393 patients. The input data includes disease, lab test, and medication codes, all binary encoded indicating their occurrence or absence. The CHF cohort is constructed by clinical experts according to the following criteria: 1) ICD-9 diagnosis of heart failure appeared in the EHR for at least two outpatient encounters, indicating consistency in clinical assessment, and 2) At least one medication was prescribed with an associated ICD-9 diagnosis of heart failure. In addition, the diagnosis date was defined as its first appearance in the record. These criteria have also been previously validated as part of Geisinger Clinical involvement in a pay-for-performance pilot study conducted by Centers for Medicare and Medicaid Services (CMS) [28]. More details could be found in [29]. A sample of EHR record segments is illustrated in Fig 1. In addition to the CHF dataset, we also evaluated based on a synthetic EHR data simulated from a de-identified real world patient dataset. The synthetic data is generated as follows: for each original patient record we randomly sample 30% to 50% of the visits in that record and drop the un-sampled visits. After subsampling, we permute patient index. Next, for each new patient record, we randomly combine it with another new record, with the event time of the second patient record being aligned to the first one. We consider such combined record as one synthetic patient record. Following this approach, we generated 3000 synthetic patients, of which 2000 are used in model training, 500 for validation, and 500 for testing. The synthetic data will serve as a benchmark for reproducing experimental results in this paper. However, since they cannot faithfully reflect real patient conditions, the performance comparison will more rely on the real world CHF data. We will also only discuss the learned patient patterns based on the results from the real world data. The basic statistics for both datasets are summarized in Table 1.

**Table 1:** Basic statistics of CHF and synthetic datasets.

Dataset	Congestive Heart Failure	Synthetic EHR Data
# patients	5, 393	3, 000
# visits	455, 106	239, 936
# events	1, 306, 685	685, 482
Avg. # of visits per patient	84.4	79.98
Avg. # of events per patient	242.3	228.49
# of unique event codes	618	618

### The CONTENT model

**Fig 2:**
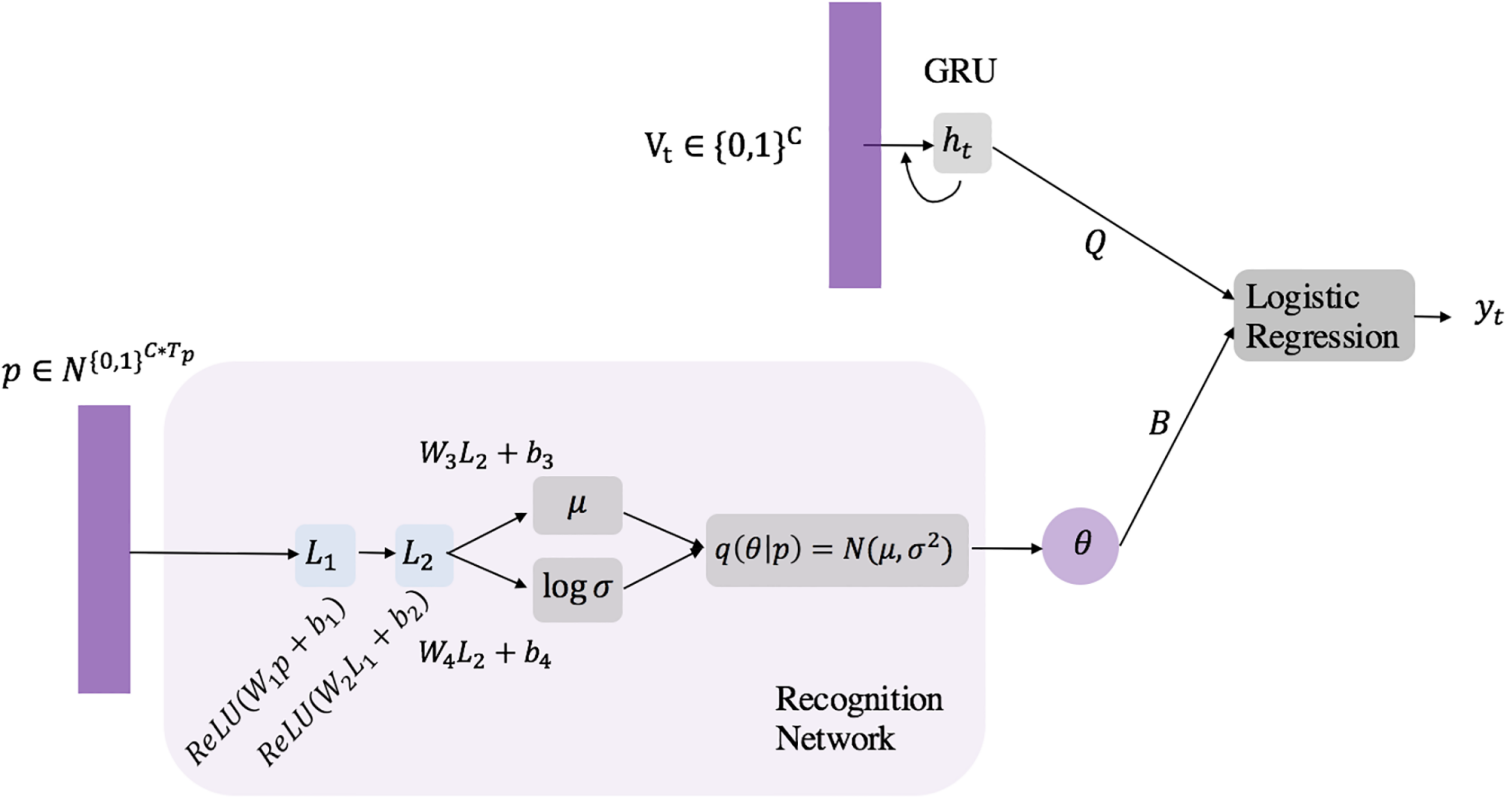
The CONTENT model.

We formalize the CONTENT model in this section. Denote *C* as the number of medical events in the EHR data and {*c*_1_, ⋯, *c*_*C*_} as the set of medical events. Each patient ***p*** makes *T*_*p*_ visits $V_{1},\cdots ,V_{T_{p}}$, where the visit ***V***_*t*_ at time *t* can be represented using a subset of medical events. Given such a structure, it is easy to draw an analogy between data in our model and language models: the set of patients can be considered as the document corpus, the EHRs of each patient can be regarded as a separate document, and each visit of a specific patient can be viewed as a paragraph in a document. Thus representing a patient as a sequence of visits is just as representing a document as a sequence of paragraphs. The difference is that in our case all events within the same visit are treated as simultaneous events. With such an analogy, the CONTENT model can be similarly constructed as a language model. We denote ***y*** = {*y*^1^, ⋯, *y*^*N*^} as the observed patient hospital admission indicators, ***h*** = {***h***^1^, ⋯, ***h***^*N*^} as the collection of RNN hidden states for all patients with $h^{p}=\left\{h_{1}^{p},\ldots ,h_{V_{p}}^{p}\right\}$ being the RNN hidden state sequence for patient ***p***. Θ is the collection of all model parameters, and *θ* is the hidden variable which represents the context vector. The hospital readmission prediction will be made based on the combination of the patient context vector and the hidden state of an RNN model. Fig 2 provides an illustration of the CONTENT model.

The CONTENT model is essentially a generative model. Its generative process is described as follows: for a particular patient ***p*** with visits $V_{1:T_{p}}$,

1. Draw patient context vector ***θ*** ∼ *N*(0, ***I***).
2. For the *t*th visit,
  - Computer hidden state ***h***_*t*_ = *GRU*(***V***_*t*−1_, ***h***_*t*−1_;***W***_*v*_, ***W***_*h*_),
  - Compute logit score $z_{t}=Q^{t}h_{t}+B_{t}^{T}\theta$, $B_{t}=\frac{1}{M_{t}}\sum b_{m}$ and *b*_*m*_ is the latent topic vector for medical code *m* in this visit; *M*_*t*_ is the number of codes in the visit.
  - Compute readmission indicator *y*_*t*_ ∼ *σ*(*z*_*t*_).

Assume that the dimension of the latent word embedding is *H*, and the dimension of topics is *N*. The parameters of the model include the word embedding matrix $W_{v}\in R^{C\times H}$, where *C* is the number of distinct words (medical events). ***W***_*h*_ is the RNN parameter set {***U***_*z*_, ***W***_*z*_, ***z***_*t*_, ***U***_*r*_, ***W***_*r*_, ***r***_*t*_} in the *GRU*(⋅) functions as defined in previous section. Here we also have $Q\in R^{H}$ and $b_{m}\in R^{N}$. Following the general auto-encoding variational Bayes model and the TopicRNN, we also use the multivariate Gaussian prior for ***θ*** to make it easier for inference. The context vector $\theta \in R^{N}$ encodes the patient’s contextual information, which can be regarded as the different clinical subtypes of the hospital readmission task.

### Model inference

To make predictions, ideally we need to maximize the log marginal likelihood:

$$\begin{array}{c} \log p(y|h,\Theta )=\log\int p(y|h,\Theta ,\theta )p(\theta )d\theta . \end{array}$$

However, directly optimizing it is intractable [30], so we adopt approximate variational inference techniques [30] to approximate it. Let *q*(***θ***) be the variational distribution that approximates the intractable posterior distribution *p*(***θ***|***y***). The log marginal likelihood could be rewritten as

$$\begin{array}{c} \log p\left(y|h,\Theta \right)=D_{KL}\left(q\left(\theta \right)|||p\left(\theta |y\right)\right)+ELBO. \end{array}$$

Here, the first term is the Kullback-Leibler (KL) divergence that measures the distance between the approximate distribution *q*(***θ***) and the true posterior *p*(***θ***|***y***). The second term is the evidence lower bound (ELBO) [31] with the following form:

$$\begin{array}{c} ELBO=E_{q(\theta )}\left[\log p\left(y|h,\theta ,\Theta \right)+\text{log}\;p\left(\theta \right)-\text{log}\;q\left(\theta \right)\right]\leq \text{log}\;p\left(y|h,\Theta \right) \end{array}$$

where the ELBO is the variational objective function to be optimized. It is a lower bound to the marginal log likelihood by positivity of the KL divergence. It therefore constitutes a principled objective for optimizing the log marginal likelihood.

Following TopicRNN [26] and the recent techniques in deep generative models [30], we formulate *q*(***θ***) as an inference network using a feed-forward neural network. The inference network takes the patient representation matrix ***p*** as the input, and then project it onto a lower dimensional subspace using a multilayer perceptron (MLP) as formulated below.

$$\begin{array}{cc} & r_{1}=ReLU\left(W_{r_{1}}p+b_{r_{1}}\right)\\ & r_{2}=ReLU\left(W_{r_{2}}r_{1}+b_{r_{2}}\right)\\ & \mu (p)=W_{\mu }r_{2}+b_{\mu }\\ & \log\sigma (p)=W_{\sigma }r_{2}+b_{\sigma }\\ & q\left(\theta |p\right)=N(\mu (p),diag\left(\sigma^{2}\left(p\right)\right))\\ & \theta ∼q(\theta |p). \end{array}$$

For each time step, we update the RNN hidden state at time stamp *t*, ***h***_*t*_, to model the sequence of visits for the patient. We do so by representing a visit at time *t* as a binary vector ***V***_*t*_ ∈ {0, 1}^*C*^, where the *i*-th entry is 1 only if *c*_*i*_ ∈ ***V***_*t*_ with *c*_*i*_ being the *i*-th distinct medical event in the dictionary. For the *T*_*p*_-th visit, the inference network only takes the patient matrix from previous visits of the patient, *p*[0: *T*_*p*_] of size *T*_*p*_, as the input. Thus, the patient matrix ***p*** is of dimension *C* × *T*_*p*_ where *T*_*p*_ is the number of the visits of the patient. The hidden state is then updated following the GRU update rule: ***h***_*t*_ = *GRU*(***V***_*t*−1_, ***h***_*t*−1_;***W***_*v*_, ***W***_*h*_). The hospital readmission indicator *y*_*t*_ at time step *t* is then computed via logistic regression using both the hidden state of the RNN ***h***_*t*_ and the patient-specific context vector $\theta :y_{t}=\sigma \left(Q^{T}h_{t}+B_{t}^{T}\theta \right)$. Note this approach is highly scalable since it does not have the bottleneck of computing the normalization constant of the softmax function as is the case in language models.

The ELBO depends on all parameters of the model, including the weight matrices of the recognition network and the recurrent neural network. The learning procedure for the CONTENT model is to estimate the optimal values of those parameters by using stochastic gradient descent (Adam) [32] with back-propagation through time.

### Clinical concept and patient embedding

The projection matrix ***W***_*v*_ can be thought of as a matrix that embeds the clinical concepts (i.e., medical events) into the low dimensional space. The context vector ***θ*** sampled from the recognition network serves as a distributed representation of the patient’s medical history. Then we can represent each patient as the concatenation of the context vector and the final hidden state vector of the RNN. In the empirical studies, we will demonstrate their representation power by clustering patients using these vector representations.

### Evaluation strategy

We assess the performance of the proposed CONTENT model on the task of CHF patient readmission prediction. Specifically, we predict whether a CHF patient who is currently in hospital will be re-admitted as “in hospital” within 30 days of his or her release from the current “in hospital” episode. Since the task is a binary classification, we choose the area under the receiver operating characteristic curve (ROC-AUC), the area under the precision-recall curve (PR-AUC), and the accuracy (ACC) as three measures. A model with higher ROC-AUC or PR-AUC is considered a better model. Advantages of AUCs as metrics are that they do not require choosing a threshold for assigning labels to scores and that they are independent of class bias in the test set.

### Model implementation

The proposed model is implemented using Theano 8.2 [33]. Code can be found in https://github.com/danicaxiao/CONTENT. RNN was implemented as a Gated Recurrent Unit (GRU). The word embedding sequences are used as inputs, and a logistic regression is applied over the hidden layer. The hyper-parameters of CONTENT and baselines are set as follows: 1) for word embedding via word2vec [25], we get word vectors of 100 dimensions. 2) the size of hidden layers of RNN is 200. Training is done through Adam at learning rate 0.001 with shuffled mini-batches of batch size 1. For model comparison, we split the data into training (4000 patients), validation (700 patients), and testing (693 patients). We train the model using the training data, optimize the parameters on validation data, and compare model performance using the out-of-sample testing strategy on testing data. The experiment was repeated 10 times and we report the average performance along with the standard deviations.

## Results

### Performance comparison of readmission predictions

Table 2 compares the prediction performance of the proposed model with several state-of-the-art baselines. The proposed CONTENT model outperforms baselines on all metrics. It is due to CONTENT incorporates both local and global contextual information, especially for the diseases that have heterogeneous manifestations such as CHF. The GRU+word2vec predicts worse than the basic GRU model. This may be due to low-dimensional concept embedding via word2vec blurs the boundary of some heterogeneous subtypes and thus causes wrong predictions. In addition, the RETAIN [34] also predicts worse than a basic GRU model. Although RETAIN adopts a sophisticated attention mechanism to set more weights on events that are considered more important, their attention strategy is not relevant to the prediction task (e.g. readmission prediction) since the attention weights in [34] are generated from only the hidden states of the GRU, while the task-related context could be ignored by this model. For Med2vec [35], we did not use the demographic information in the original paper in order to keep a fair comparison. The Med2vec takes advantage of the hierarchical information of the EHR data, and thus is a better representation method than word2vec and gaining better results.

**Table 2:** Performance comparison on CHF data. CONTENT outperforms Word2vec+LR, Med2vec+LR, GRU, GRU+Word2Vec, and RETAIN on different performance metrics.

Method	PR-AUC	ROC-AUC	ACC
Word2vec+LR	0.3445±0.0204	0.5360±0.0246	0.6828±0.0120
Med2vec+LR	0.3836±0.0149	0.5937±0.0120	0.6915±0.0095
GRU	0.3862±0.0136	0.5998±0.0124	0.6856±0.0082
GRU+Word2Vec	0.3430±0.0157	0.5616±0.0157	0.6731±0.0091
RETAIN	0.3720±0.0148	0.5707±0.0140	0.6814±0.0111
CONTENT	0.3894±0.0153	0.6103±0.0130	0.6934±0.0090

In Table 3 we compare the prediction performance based on a set of synthetic data generated from a real generic patient cohort. During data generation, for each raw sequence of events, we dropped randomly sampled 30%−50% events, perturbed the time information for each visits, combined it with another subsampled sequence of events. The generation procedure effectively introduced lots of missing information, noise and anomaly. Results show that the proposed CONTENT model again outperforms most baselines due to it models patient representations and predict readmissions not only based on the RNN states but also on the topics. As the topics are exchangeable and globally modeled as a context, the CONTENT would be less impacted by some missing visits, noise and perturbed time information. However, when comparing with RETAIN, the proposed model gains much better PR-AUC since the precision is much higher, but slightly worse ROC-AUC since the attention model in RETAIN improves prediction accuracy in general.

**Table 3:** Performance comparison on synthetic data. CONTENT outperforms Word2vec+LR, Med2vec+LR, GRU, GRU+Word2Vec, and RETAIN on different performance metrics.

Method	PR-AUC	ROC-AUC	ACC
Word2vec+LR	0.5155±0.0021	0.6040±0.0188	0.6229±0.0179
Med2vec+LR	0.5906±0.0057	0.6884±0.0044	0.7170±0.0087
GRU	0.5929±0.0100	0.6881±0.0048	0.7141±0.0040
GRU+Word2Vec	0.5907±0.0174	0.6836±0.0031	0.7117±0.0045
RETAIN	0.5525±0.0005	0.6927±0.0001	0.7310±0.0001
CONTENT	0.6011±0.0191	0.6886±0.0074	0.7170±0.0069

### Clustering of patient patterns

**Fig 3:**
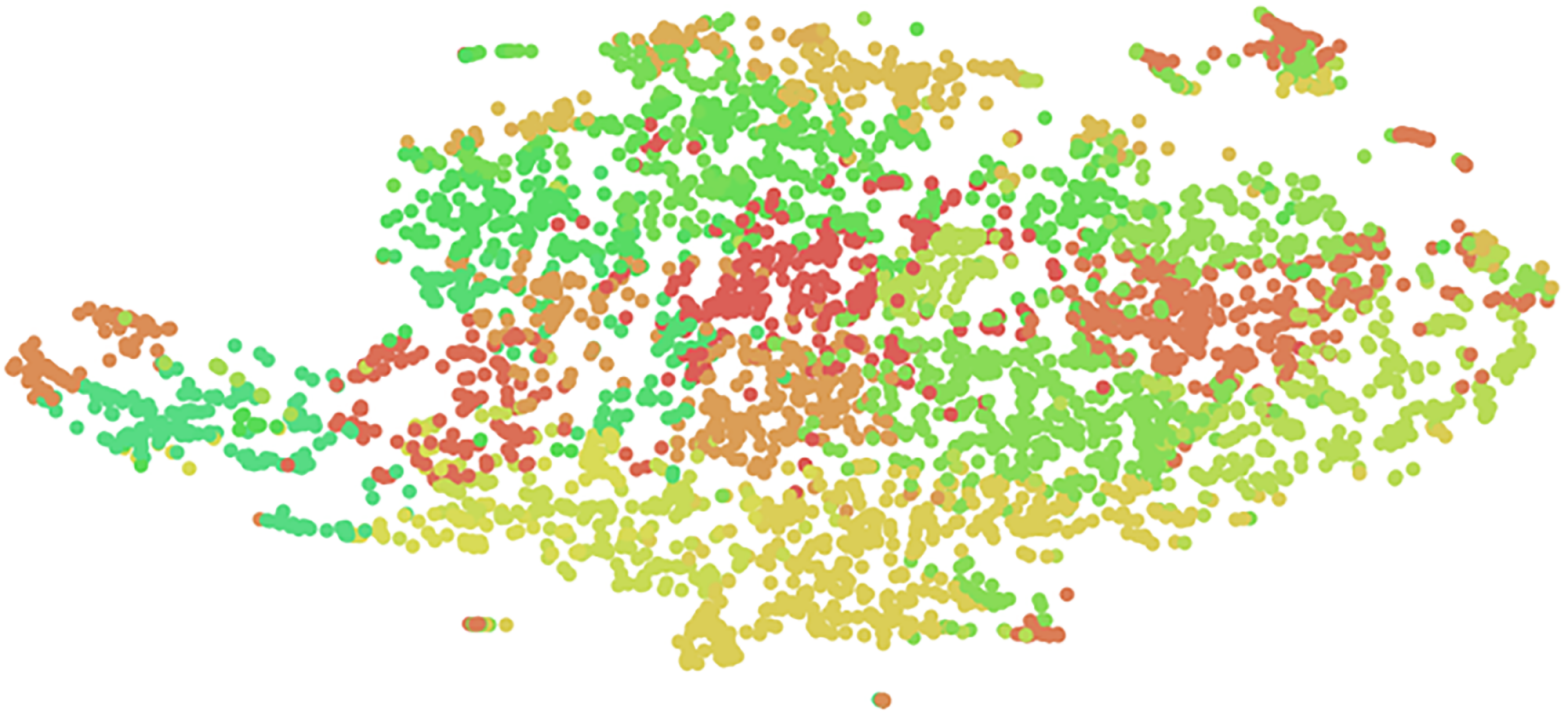
Clustering of patient representations.

As we explained in the model inference section, to gain understanding of the learned patient representations, we concatenate the topical context vector ***θ*** and the final hidden state of RNN as the patient-specific vector representation. These vectors are then used to cluster the CHF cohort into patient subgroups with more homogeneous latent patterns. To be specific, we apply k-means algorithm and set k = 20 to generate 20 subgroups. The clustering result is plotted in Fig 3.

**Fig 4:**
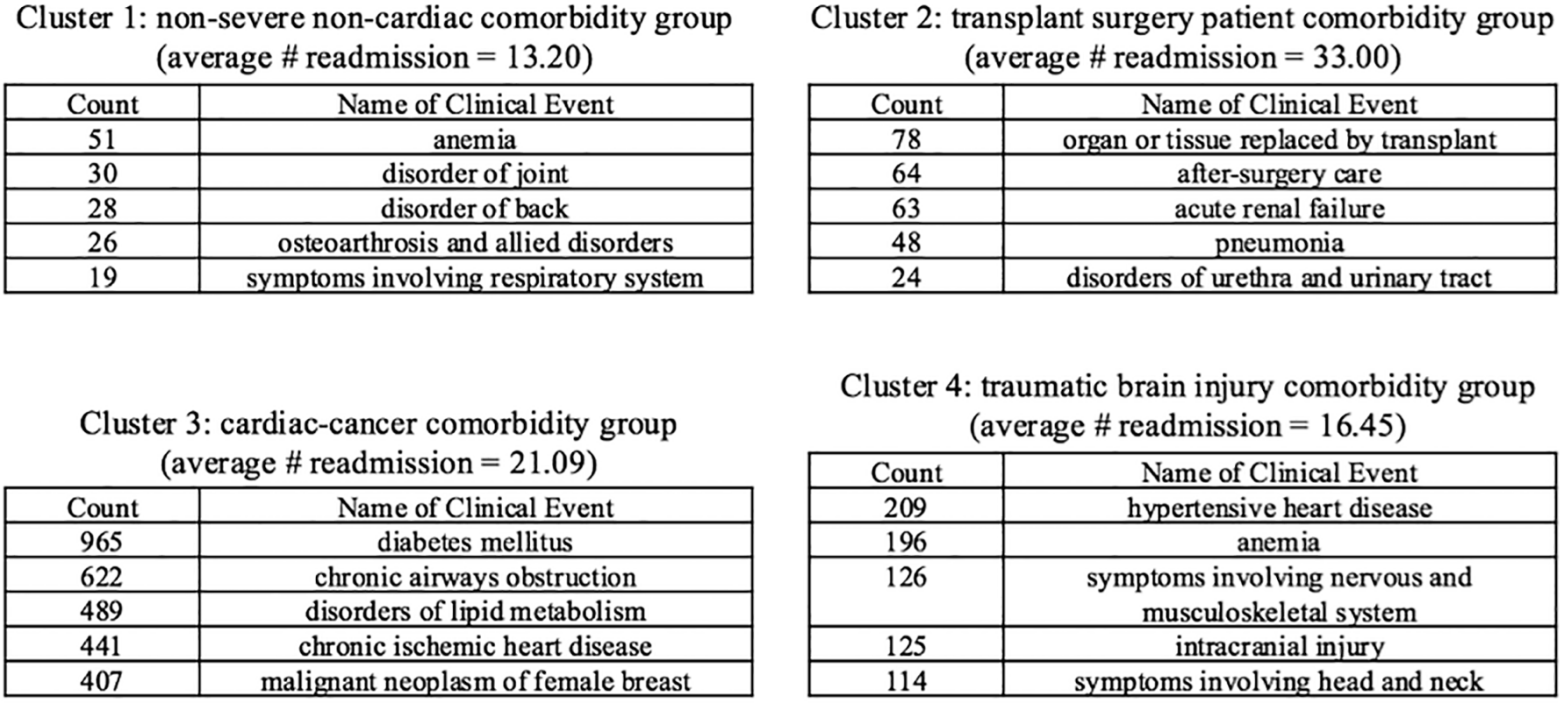
Top clinical events for selected clusters.

To take a closer look at the learned subgroups, we pick 4 clusters out of the 20 clusters. To quantitatively evaluate their differences, we calculated the average number of readmission for each cluster. In addition, we also make qualitative evaluation by analyzing the top clinical events ranked by their counts in the cluster. Note that we omit the top three common events shared by all CHF patients, including 1) essential hypertension: a major risk factor of CHF, 2) cardiac dysrhythmia: a condition about irregular heart rhythm or abnormal heart rate, and if long-term impending, could indicate higher likelihood of CHF-related hospital readmission, and 3) heart failure. These events demonstrate the commonalities of the CHF condition manifestations. We omit them in order to focus more on the cluster-specific clinical patterns. The results are listed in Fig 4.

Combine the top clinical patterns and the average count of readmissions, we find that the clusters may represent different CHF comorbidity subgroups where comorbidity conditions serve as context and would impact risks of readmissions. We studied literature and derived the most likely interpretations for the clusters as explained below.

Cluster 1 (in orange) probably relates to the comorbidity group where patients have non-severe non-cardiac conditions. For example, the top event anemia is known to be a common condition among the non-cardiac comorbidity group, with a prevalence ranging from 4% to 55% [36]. In addition, other top events, e.g. the disorders of back, disorders of joint, and osteoarthritismainly occur among senior people in the non-cardiac CHF comorbidity group as discussed in [37]. Moreover, the concomitant symptoms affecting respiratory system were also discussed in literature and considered very common to CHF patients [37]. Due to these comorbidity conditions are not critical, under such context this cluster has low readmission on average.

As a contrast, most of the top conditions in Cluster 3 (in green) are cardiac comorbidities that directly relate to the presence of CHF. In addition, many patients in this cluster were diagnosed as “malignant neoplasm of female breast (breast cancer)”. Literature indicates the comorbid of CHF would become a risk factor for poor outcomes for breast cancer, adversely impact the cancer treatments [38], and thus could lead to more frequent hospital readmissions.

In addition, we find Cluster 4 (in yellow) quite interesting as many CHF patients have the following events “intracranial injury” and “symptoms involving head and neck”. We suspect that for this group, the readmission might be due to the injuries rather than CHF itself. While the injuries could also be caused due to CHF related conditions, for example, the comorbid vision problem (e.g. cataracts) of CHF, the comorbid hypertensive heart disease, or the prevalence of various neurological disorders such as the event “symptoms involving nervous and musculoskeletal system” indicates [39].

Last, we also find Cluster 2 (in red) quite special as many patients have received organ or tissue transplant surgeries. It is reasonable to believe transplant surgeries relate to high risks of readmission for CHF patients. For CHF patients received cardiac transplantation, they were often considered at advanced stage with severe dysfunctions. This can be inferred from contaminant acute diseases, such as pneumonia and acute renal failure, as well as disorders of urethra and urinary tract, a common after surgery disorder. Moreover, CHF could be onset after transplant surgeries, e.g. the comorbid CHF after hematopoietic cell transplantation [40].

## Discussion

This study presents CONTENT, a deep model that learns distributed patient representation from the EHR data and performs prediction for the 30-day readmissions. The CONTENT incorporates global context by capturing latent topics via a recognition network and uses global context as additional bias to the output layer of an RNN model, so that the RNN can focus its modeling capacity on the local context. With this design, the proposed model achieves more accurate prediction results than the state-of-the-art baselines.

The importance of learning both local and global context from analyzing the learned clusters are two-fold: 1) although CHF patients share many commonalities, e.g. having hypertension and cardiac dysrhythmia, their risks of readmission can vary due to different local or global contextual information. For example, patients recently received heart or cell transplantation surgery have high risks of readmission, thus need more frequent follow-ups after last discharge. Another example, if patients only have non-severe non-cardiac comorbid conditions, their risks of readmission would be lower than other groups. 2) although the readmission prediction is based on CHF cohort, the patients may be readmitted due to some other reasons, which could become confounding factors here. For example, patients are readmitted due to comorbid traumatic brain injury, which can be induced by CHF comorbid conditions or other reasons.

What is worth mentioning is in this work we did not explicitly perform any missing value imputation for the input clinical events. The observation that time intervals between two visits are irregular is a very common phenomenon in healthcare and clinical setting. Since most EHR data are not missing at random (NMAR) [41–43], it is challenging for existing imputation methods to be used on EHR data. Our CONTENT model not explicitly aims for solving the problem of missing values. However, it does implicitly decrease the impact brought by missing values by capturing the global context using topics. In future work we can also employ dropout techniques in the model. The dropout technique is essentially equivalent to randomly removing some visits or codes. So the final model will be more robust to missing visits.

To summarize, the CONTENT model not only learns more accurate patient representation and thus leads to better prediction performance, but also generates interpretable representations that could be used to cluster patients into more homogeneous patient subgroups. Analyzing the top clinical features in each subgroup provide interpretability and help us gain better understanding of CHF comorbidity and various reasons and risks of 30-day readmissions for CHF patients.

The limitation of this work is that we only use clinical events as original input features. However, unlike previous models that are designed to only take sequence of events as input features, the proposed model can be extended to extract better global context from generic form of inputs, e.g. patient profile or other side information. This will be one future direction of extension.

## Conclusion

In this paper, we propose CONTENT, an end-to-end deep sequential predictive model that embeds local and global contextual information via RNN and a topic model based recognition network, respectively. We evaluated the model with hospital readmission prediction task on a cohort of CHF patients. CONTENT outperforms baseline and can also explicitly generates interpretable subgroups to improve understanding of heterogeneous readmission risks among CHF cohort. Future work includes applying the model to different cohorts to show the generality of the approach. We will also include side information for generating better global context.

## Supporting information

S1 Data The simulation data used in the experiment. S1_Data.txt is the simulation data used in the experiment and the implementation code can be found at https://github.com/danicaxiao/CONTENT.
(ZIP)
